# Dietary Cholesterol Intake Is Not Associated with Risk of Type 2 Diabetes in the Framingham Offspring Study

**DOI:** 10.3390/nu10060665

**Published:** 2018-05-24

**Authors:** Siyouneh Baghdasarian, Hsuan-Ping Lin, Richard T. Pickering, Melanie M. Mott, Martha R. Singer, M. Loring Bradlee, Lynn L. Moore

**Affiliations:** Department of Medicine Section of Preventative Medicine and Epidemiology, Boston University School of Medicine, Boston, MA 02118, USA; siyob@bu.edu (S.B.); hpl1201@bu.edu (H.P.L.); rtpicker@bu.edu (R.T.P.); melaniemmott@yahoo.com (M.M.M.); msinger@bu.edu (M.R.S.); lbradlee@bu.edu (M.L.B.)

**Keywords:** dietary cholesterol, eggs, type 2 diabetes mellitus, fasting glucose, prospective study

## Abstract

Identification of diet and lifestyle risk factors for prevention of type 2 diabetes mellitus (T2DM) is of great importance. The specific role of dietary cholesterol (DC) in T2DM risk is unclear. This study uses data from 2192 Framingham Offspring Study subjects to estimate the effects of DC alone and in combination with markers of a healthy diet and other lifestyle factors on fasting glucose and risk of T2DM or impaired fasting glucose (IFG) over 20 years of follow-up. Dietary data were derived from two sets of three-day food records. Statistical methods included mixed linear regression and Cox proportional hazard’s modeling to adjust for confounding. There were no statistically significant differences in glucose levels over 20 years of follow-up across DC intake categories (<200, 200–<300, and ≥300 mg/day) and no increased risk of T2DM/IFG associated with higher intakes. The HR for T2DM/IFG associated with consumption of ≥300 mg/day of DC was 0.87 (95% CI: 0.68–1.10). In contrast, subjects with lower intakes of fish, whole grains, and fiber had higher T2DM/IFG risk. DC consumption was not associated with fasting glucose levels or risk of T2DM/IFG over 20 years of follow-up.

## 1. Introduction

Fasting blood glucose levels in healthy individuals are maintained within a relatively narrow physiological range. Type 2 diabetes (T2DM) is diagnosed when fasting glucose rises above 126 mg/dL (7 mmol/L). Impaired fasting glucose (IFG) is diagnosed when fasting glucose levels reach above the normal range of 100 mg/dl but below the diagnostic cut-off (≥126 mg/dL) value for T2DM [1]. 

Based on the most recent Centers for Disease Control (CDC) report, 30.3 million Americans are estimated to have diabetes, with 90–95% of that figure having T2DM, and 7.2 million being undiagnosed [2]. T2DM and IFG are important risk factors for cardiovascular disease [3,4], making the prevention of these disorders a critical priority for the health care system. 

Diet and other lifestyle factors have been identified as important modifiable risk factors for T2DM and IFG [5]. While evidence suggests that healthy dietary factors including fruits and vegetables, whole grains, fiber, and fish seem to lower the risk of T2DM and IFG [6,7], other factors such as the macronutrient composition of the diet [8,9,10,11] and dietary cholesterol (DC) are less well understood. In recent years, a number of studies have explored the association between egg consumption and risk of T2DM but the results of these studies have been inconsistent [12,13,14,15]. Prospective data examining the relation between DC intake and risk of T2DM or IFG are limited.

Beginning in the 1960s, United States Dietary Guidelines recommended limiting intake of DC to no more than 300 mg per day for the prevention of cardiovascular disease [16,17]. These restrictions were removed in the 2015 Guidelines, although some questions remain about the effects of DC on glucose regulation [18]. Some earlier studies have suggested that excess DC intake among individuals with prevalent diabetes may have adverse effects on both lipids and glucose [19,20] while an analysis of healthy subjects in the Iowa Women’s Health Study found that higher DC intake was linked with a higher risk of T2DM [21]. 

The current study aims to examine the effects of DC on glucose levels and the risk of IFG and T2DM among 2192 adults in the Framingham Offspring Study (FOS) who were normoglycemic at baseline and followed over a period of up to 20 years. Additionally, potential modification by selected markers of a healthy diet and lifestyle factors such as physical activity is also explored. 

## 2. Materials and Methods 

### 2.1. Study Population

The FOS is a longitudinal cohort composed of the offspring of subjects from the original Framingham Heart Study and their spouses. It began in 1971 and included 5135 subjects at exam 1. Examination visit 2 followed 8 years later and subsequent exams have continued at four-year intervals [22]. At each exam, each subject’s medical history and lifestyle habits were assessed and measures of urine, blood chemistries, blood pressure, and body fat were collected. Subjects were also asked to report any disease or conditions that had developed since their last visit. 

Subjects who met the following criteria were included in these analyses: (1) had baseline DC intake measurements available from dietary records at 35–64 years of age; (2) had complete data for fasting blood glucose and (3) complete data for all confounders included in the final models (age, sex, cigarettes per day, grams of alcohol per day, baseline BMI, percent energy from carbohydrates, whole grain and dairy intake). Subjects with prevalent type 1 or type 2 diabetes or prevalent cancer (except non-melanoma skin cancer) were excluded, leaving a total of 2192 individuals for these analyses. 

### 2.2. Dietary Assessment

Three-day diet records were collected during exam cycles 3 and 5, yielding a total of six days of diet records. Information from the dietary records was entered into Nutrition Data System (NDS), a nutrient calculation program from the University of Minnesota [23]. Data from all foods and beverages consumed were used to calculate each individual’s daily intake of nutrients, including calories, macronutrients, and micronutrients. Dietary cholesterol and other nutrients were derived from the mean intake of all-available days of dietary records. Subjects who were too young to be included in the study population or who were missing dietary data at exam 3 were allowed to enter at exam 5 if within the correct age range. 

### 2.3. Assessment of Incident Type 2 Diabetes and Impaired Fasting Glucose

Subjects were diagnosed with T2DM who met one of the following conditions: (a) non-fasting glucose of 200 mg/dL or higher; (b) fasted for 10 hours or more and had a glucose level of 126 mg/dL or higher; (c) had a confirmed history of treated diabetes (with oral hypoglycemic medication or insulin); or (d) reported a diagnosis of diabetes and developed definite diabetes at the next exam without gaining 7% or more of body weight between exams. Subjects who had a glucose level of 100–125 mg/dL but who did not meet the above criteria for T2DM were diagnosed as having IFG. 

### 2.4. Potential Confounding Variables

In these analyses, only those factors that were found to be confounders of the relation between DC and T2DM or IFG were included in the final models. These factors included age, sex, cigarette smoking, alcohol intake, BMI, and other dietary factors. Only percent of energy from carbohydrates and servings per day of whole grains and dairy were dietary confounders of the effects. Cigarette smoking and alcohol intake were assessed at every exam by interview. Height and weight were measured at each exam using a standard beam balance scale. Exam-specific BMI was calculated using each exam’s weight measurement (in kilograms) divided by the mean of all measured heights prior to age 60. This approach was used to minimize random measurement error as well as the effects of age-related height loss on BMI.

### 2.5. Statistical Analysis

DC intake for each subject was classified into one of three categories of intake: low (<200 mg/day), moderate (200–<300 mg/day), or high (≥300 mg/day). These cutoff values were selected using sensitivity analyses and were also designed to include 300 mg/day as the cutoff value used in previous dietary guidelines for DC intake. For dietary pattern analyses, DC intake and other food intakes were dichotomized and cross-classified using the following cutoff values: DC, <300 versus (vs.) ≥300 per day; fish, <1 vs. ≥1 ounce-equivalents per week; fiber, <15 vs. ≥15 g per day; whole grains, <0.5 vs. ≥0.5 ounce-equivalent per day; fruits and vegetables, <3 vs. ≥3 cup-equivalents per day. In addition, DC intake was also cross-classified with the following other risk factors for diabetes: physical activity, tertile 1 vs. tertiles 2–3; alcohol, 0, 1–<24, and ≥24 g/day (man) and 0, 1–<12, and ≥12 g/day (women); cigarette smoking, none smokers vs. smokers (non-smokers are no current smoker or <1 cigarette/day, smokers smoke 1 or more cigarettes/day); and BMI, <25, 25–<30, and ≥30 kg/m^2^. 

The association between DC intake and change in glucose over time was evaluated using mixed linear regression modeling for repeated measures data. These models included both fixed and random effects for the various potential confounding factors. The models used an unstructured covariance assumption. Subjects who developed T2DM at an exam after baseline were excluded from the point of diagnosis.

The rates of T2DM and IFG were calculated for each category of dietary cholesterol intake. Person-years of follow-up time were calculated from time of the final dietary assessment (generally exam 5) to the first of the following events: occurrence of T2DM or IFG, loss to follow up, date of last exam, or date of death. The incidence rates of T2DM or IFG were calculated by dividing the total number of events (T2DM and IFG) by the total number of person-years in a given exposure category; rates were expressed per 1000 person-years. Cox proportional hazards were used to estimate adjusted hazards ratios for the first occurrence of T2DM or IFG. There were no violations of the proportional hazards assumptions in the models. 

All analyses were controlled for age (years), sex, cigarettes smoked per day, alcohol intake (grams per day), BMI, percent of energy from carbohydrates, and intakes of whole grains and dairy. Additional variables, such as education level, physical activity, pack-years of smoking, and other dietary factors that were explored but not found to confound the relationship of DC intake with T2DM and IFG risk were excluded from the final models. All statistical analyses were carried out using SAS version 9.3 (SAS Institute Inc, Cary, NC, USA). 

## 3. Results

The baseline characteristics of the subjects according to category of DC intake are shown in Table 1. In these unadjusted models, subjects with the highest DC intakes tended to have a higher anthropometric measures of body fat. They also smoked more cigarettes per day, had higher intakes of alcohol, and had higher intakes of fat and saturated fat but lower intakes of carbohydrates and whole grains. Of note, male subjects are clustered in highest DC intake group (65%) and females in the lowest DC intake group (75%). 

**Figure 1:**
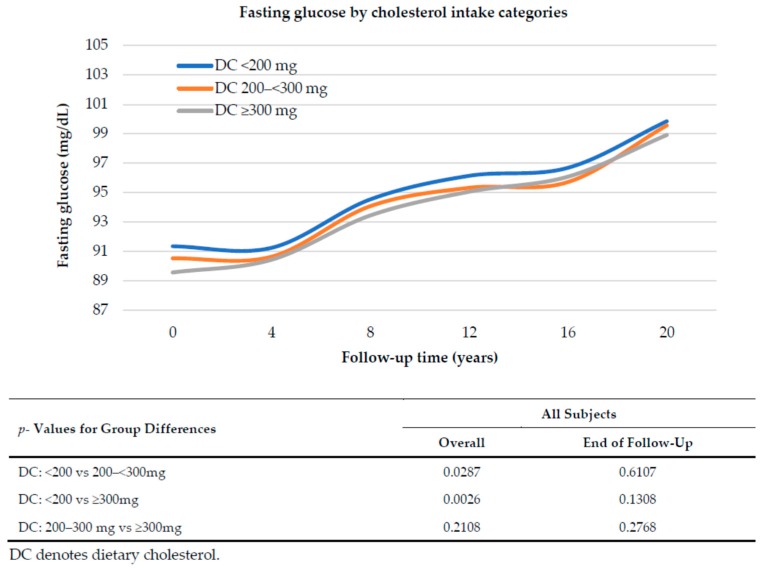
Fasting glucose over 20 years of follow up associated with dietary cholesterol intake category. Mean glucose levels are adjusted for sex, age, pack-years of smoking, BMI, percent of energy from carbohydrates, whole grains and dairy intake, using mixed linear regression models.

Figure 1 explores the relation between dietary cholesterol intake and fasting glucose level over 20 years of follow up, adjusting for age, sex, pack-years of smoking, BMI, percent of energy from carbohydrates, and intakes of whole grains and dairy. Mean fasting glucose levels rose over time regardless of the amount of dietary cholesterol consumed. Overall, there was a tendency for subjects with higher DC intakes to have slightly lower fasting glucose levels but there were no statistically significant differences at the end of follow up in fasting glucose. Further, there was no indication of greater increases in fasting glucose levels among those with higher DC intakes.

Cox proportional hazard’s models were used to evaluate the risk of developing incident T2DM or IFG over up to 20 years of follow up (Table 2). There is a trend toward higher rates of incident IFG or T2DM with increasing DC intake (15.1, 18.1, and 19.5 cases per 1000-person years in the low, moderate, and high intake groups, respectively). After adjusting for confounding, however, there was no indication of an increase in risk of T2DM/IFG. In fact, the highest DC intake group had a non-statistically significant 13% reduction (95% CI: 0.68–1.10) in risk. Similar results were seen for males and females. 

While there was no overall association between higher DC intake and risk of T2DM/IFG, it is possible that these effects might differ based on other factors in an individual’s diet. Therefore, in Table 3, we examined the effect of DC (<300 vs. ≥300 mg per day) in combination with other dietary factors previously associated with diabetes risk such as intakes of fish, fiber, whole grains, and fruit and vegetables (Table 3). In all cases, we used the dietary pattern that was deemed *a priori* to be healthiest (e.g., lower DC plus higher fish intake) as the referent category. It was evident that lower (vs. higher) intakes of fish, fiber, and whole grains were associated with 25–34% higher risks of T2DM/IFG. In contrast, higher DC intake led to no increase in risk of T2DM/IFG, regardless of other dietary factors. 

Finally, in Table 4, we evaluated whether the effects of DC were modified by other lifestyle factors such as physical activity, alcohol consumption, smoking, or baseline BMI. Both smoking and higher BMI were strong independent risk factors for T2DM/IFG. For example, smokers had a 73% increased risk of T2DM/IFG (HR: 1.73; 95% CI: 1.38–2.16) that was independent of DC intake. In contrast, a higher DC intake among non-smokers led to no increase in risk of T2DM/IFG. The combined exposure category for each analysis represents those subjects with higher DC intakes plus one of the other risk factors such as low activity levels or obesity. In each case, there was no indication that higher DC intake strengthened the adverse effects of other risk factors. For example, while obese individuals who had high DC intakes had a 2.81-fold increased risk of T2DM/IFG (compared with individuals having low DC and normal weight), this risk was actually lower than that found among obese subjects who had lower DC intakes. Thus, there was no support for an adverse effect of consuming 300 mg/day or more of DC, regardless of other lifestyle factors. 

## 4. Discussion 

With 20 years of follow up in these analyses from the Framingham Offspring Study, there was no association between fasting glucose and DC intake. After carefully controlling for confounding factors, fasting glucose tended to be slightly higher throughout follow up among those whose DC intake was below 200 mg per day. In addition, there was no association been DC intakes at or above 300 mg per day and risk of T2DM or IFG over the follow up period. In these analyses, we also explored whether the impact of DC intake on T2DM or IFG might be modified by other dietary or lifestyle factors. The intakes of fish, whole grains, fiber, and light to moderate alcohol intake were generally protective while smoking and excess body weight had adverse effects on risk of T2DM and IFG. In these stratified analyses, there was no independent association between DC and these metabolic outcomes. 

Few previous prospective studies have examined the direct relation between DC intake and T2DM or IFG. Most frequently, eggs have been used as a surrogate for dietary cholesterol intake and the effects on diabetes risk have been variable, with some studies suggesting an adverse effect and others finding eggs to be inversely associated with T2DM risk [12,15,24,25,26,27]. A recent review concluded that any apparent adverse effect of egg consumption on either CVD or diabetes risk was most likely due to confounding by other dietary factors [28]. Three recent meta-analyses have been published and all found that the slightly higher risks of T2DM associated with egg consumption were limited to U.S. studies [29,30,31]. In one meta-analysis, studies deemed to be of higher quality were more likely to be null. It is possible, however, that the adverse effects found in U.S. studies could be due to differences in overall dietary patterns associated with egg intake leading to residual confounding.

While eggs are a major source of dietary cholesterol, meat is also an important source, at least in the U.S. For this reason, we chose to examine total DC intake (from all sources) and risk of T2DM and IFG in the current study. A few studies have similarly examined total DC. Data from a prospective analysis of the Women’s Health Study found higher risks of T2DM in all but the lowest quintile of DC intake, measured with a semi-quantitative food frequency questionnaire (FFQ) [32]. In the Iowa Women’s Health Study, a 17% increased risk of T2DM was found in the highest quintile of DC intake but this was attenuated after controlling for dietary fats [21]. In a prospective study from Japan, investigators found that DC intake was not associated with T2DM risk in men and inversely associated with risk in women [33].

While there is no clear mechanism linking DC intake with T2DM or IFG, it is possible that DC may promote higher systemic [34] or liver-specific [35] inflammation which may promote insulin resistance. Additionally, high DC may have effects on pancreatic islet function that reduce the ability to respond to glucose [36]. 

There are a number of important strengths of the current analyses. In particular, the detailed assessment of dietary intake from diet records provides both a more accurate measure of DC intake as well as other potential dietary confounders. In addition, the Framingham Studies have carefully measured indicators of other lifestyle-related factors as well as standardized and validated assessments of the outcomes of interest. The study is limited by the homogeneous population of predominantly Caucasian subjects. In addition, there were few subjects with very high intakes of dietary cholesterol, particularly among women. 

## 5. Conclusions

The findings from this study do not support the need to limit DC intake for the prevention of T2DM or IFG.

## Figures and Tables

**Table 1:** Baseline characteristics of Framingham Offspring Study subjects according to dietary cholesterol intake.

Baseline Characteristics	Dietary Cholesterol Intake/Day			*p*-Values ^1^
	<200 mg	200–<300 mg	≥300 mg	
	(*N* = 762)	(*N* = 776)	(*N* = 654)	
	mean (s.d.)			
Age (years)	50.0 (8.2)	48.8 (8.3)	47.6 (8.1)	<0.0001
Fasting glucose (mg/dL)	90.7 (8.2)	90.8 (8.1)	91.0 (8.0)	0.7518
BMI (kg/m^2^)	25.4 (4.3)	26.0 (4.3)	26.7 (4.4)	<0.0001
Waist circumference (inches)	33.3 (5.4)	34.8 (5.5)	36.3 (5.3)	<0.0001
Cigarettes (pack-years)	12.3 (17.9)	14.4 (19.3)	15.9 (19.9)	0.0010
Physical activity (hours/day)	12.1 (7.5)	12.3 (8.5)	13.0 (8.7)	0.0764
Energy intake (kilocalories/day)	1586 (369.4)	1908 (464.0)	2288 (537.7)	<0.0001
Protein (% of energy)	16.6 (3.3)	17.0 (3.3)	16.8 (3.0)	0.0354
Carbohydrates (% of energy)	50.0 (7.9)	45.3 (7.2)	42.4 (6.8)	<0.0001
Total fat (% of energy)	32.5 (6.6)	35.6 (5.9)	38.1 (5.6)	<0.0001
Saturated fat (% of energy)	10.6 (2.8)	12.1 (2.7)	13.6 (2.6)	<0.0001
Dietary fiber (grams/day)	15.6 (6.7)	15.9 (6.2)	16.5 (5.9)	0.0445
Fruits and vegetables (cups/day)	3.1 (1.5)	3.1 (1.4)	3.1 (1.4)	0.6954
Whole grains (oz eq/day)	0.68 (0.8)	0.59 (0.7)	0.55 (0.6)	0.0035
Lean meat, poultry, fish (oz eq/day)	2.92 (1.6)	3.66 (1.9)	4.05 (2.2)	<0.0001
Eggs (oz eq/week)	1.12 (1.3)	2.3 (1.6)	5.2 (2.8)	<0.0001
Alcohol (grams/day)	7.85 (11.7)	11.71 (17.5)	13.8 (18.5)	<0.0001
Sex (% male)	24.9	44.2	65.3	<0.0001
Education (% college graduate)	33.1	35.7	38.5	0.0325

Ounce equivalents denotes oz eq. ^1^ *p*-values for continuous variables from ANOVA; *p*-values from categorical values from Mantel-Haenszel chi-square tests.

**Table 2:** Rates and adjusted hazard ratios for incident T2DM or IFG according to dietary cholesterol intake.

	All Subjects					
Exposure Category	*N*	Person Years	Events ^1^	Rate/1000PY	HR ^2^	95% Confidence
DC <200	762	11739	177	15.08	1.00	–
DC 200–<300	776	11683	212	18.15	1.01	0.82–1.24
DC ≥300	654	9734	190	19.52	0.87	0.68–1.10

DC denotes dietary cholesterol (mg), HR denotes hazard ratio; CI denotes confidence interval, PY denotes person years. ^1^ Events include incident cases of type 2 diabetes mellitus or impaired fasting glucose; ^2^ Adjusted for sex, age, pack-years of smoking, baseline BMI, percent of energy from carbohydrates, whole grains and dairy intake.

**Table 3:** Rates and adjusted hazard ratios for T2DM or IFG according to combined intakes of dietary cholesterol and other dietary factors.

	All Subjects					
Combined Intakes	*N*	Person Years	Events ^1^	Rate/1000PY	HR ^2^	95% CI
DC <300, Fish ≥1 svg	718	11,370	175	15.39	1.00	–
DC <300, Fish <1 svg	820	12,053	214	17.76	1.25	1.03–1.53
DC ≥300, Fish ≥1 svg	346	5230	98	18.74	0.96	0.74–1.25
DC ≥300, Fish <1 svg	308	4504	92	20.43	0.98	0.75–1.28
DC <300, Fiber ≥15 g	724	11,061	170	15.37	1.00	–
DC <300, Fiber <15 g	814	12,362	219	17.72	1.23	0.98–1.53
DC ≥300, Fiber ≥15 g	360	5597	104	18.58	0.93	0.71–1.20
DC ≥300, Fiber <15 g	294	4137	86	20.79	1.03	0.77–1.39
DC <300, Whole Grain ≥0.5 svg	682	10,668	152	14.25	1.00	–
DC <300, Whole Grain <0.5 svg	856	12,755	237	18.58	1.34	1.08–1.65
DC ≥300, Whole Grain ≥0.5 svg	267	4071	75	18.42	0.95	0.71–1.28
DC ≥300, Whole Grain <0.5 svg	387	5663	115	20.31	1.09	0.84–1.42
DC <300, Fruits & Vegetables ≥3 svgs	713	11,067	174	15.72	1.00	–
DC <300, Fruits & Vegetables <3 svgs	825	12,355	215	17.40	1.09	0.88–1.34
DC ≥300, Fruits & Vegetables ≥3 svgs	320	4888	93	19.03	0.91	0.70–1.20
DC ≥300, Fruits & Vegetables <3 svgs	334	4846	97	20.02	0.91	0.68–1.20

DC denotes dietary cholesterol (mg), HR denotes hazard ratio; CI denotes confidence interval, PY denotes person years, svg denotes serving. ^1^ Events include incident cases of type 2 diabetes mellitus or impaired fasting glucose; ^2^ Adjusted for age, sex, pack-years of smoking, % of energy from carbohydrates, whole grains (except in whole grains model) and dairy intake.

**Table 4:** Rates and adjusted hazard ratios for T2DM or IFG according to combined intakes of dietary cholesterol and other factors.

Exposure Categories ^1^	All Subjects					
	*N*	Person Years	Events ^2^	Rate/1000PY	HR ^3^	95% CI
DC <300, Activity (moderate/high)	1005	15,273	252	16.50	1.00	–
DC <300, Activity (low)	533	8150	137	16.81	1.10	0.89–1.35
DC ≥300, Activity (moderate/high)	441	6547	124	18.94	0.87	0.69–1.10
DC ≥300, Activity (low)	213	3188	66	20.70	0.93	0.70–1.24
DC <300, Non-drinker	470	7140	126	17.65	1.00	–
DC <300, Light-moderate drinker	762	11,728	168	14.33	0.78	0.61–0.99
DC <300, Heavier drinker	306	4554	95	20.86	1.04	0.78–1.38
DC ≥300, Non-drinker	166	2414	48	19.89	0.78	0.55–1.11
DC ≥300, Light-moderate drinker	335	5030	93	18.49	0.75	0.56–1.01
DC ≥300, Heavier drinker	153	2290	49	21.40	0.82	0.57–1.18
DC <300, Non-smoker	1204	18,707	276	14.75	1.00	–
DC <300, Smoker	334	4716	113	23.96	1.73	1.38–2.16
DC ≥300, Non-smoker	482	7338	128	17.44	0.88	0.70–1.11
DC ≥300, Smoker	172	2396	62	25.88	1.32	0.98–1.77
DC <300, BMI <25	744	12,174	108	8.87	1.00	–
DC <300, BMI 25–<30	574	8427	175	20.77	2.08	1.62–2.67
DC <300, BMI ≥30	220	2822	106	37.57	3.85	2.93–5.07
DC ≥300, BMI <25	238	3815	42	11.01	1.04	0.72–1.50
DC ≥300, BMI 25–<30	301	4421	99	22.39	1.92	1.42–2.59
DC ≥300, BMI ≥30	115	1498	49	32.72	2.81	1.96–4.83

DC denotes dietary cholesterol (mg); HR denotes hazard ratio; CI denotes confidence interval, PY denotes person years. ^1^ For activity, low = tertile 1, moderate/high = tertiles 2–3; for alcohol, non-drinker = no current alcohol, light-moderate drinker = 1–<24 g/day, for men and 1–<12 g/day, for women, heavier drinker ≥24 g/day for men and ≥12 g/day for women; for smoking, non-smokers = no current smoking or <1 cigarette/day, smoking = 1 or more cigarettes/day; ^2^ Events include incident cases of type 2 diabetes mellitus or impaired fasting glucose; ^3^ Adjusted for age, sex, pack-years of smoking, % of energy from carbohydrates, whole grains, and dairy intake.
